# Multi-omics analysis reveals Angelica dahurica radix extract alleviates migraine in rats via gut microbiota-metabolome-gut-brain axis regulation

**DOI:** 10.3389/fphar.2025.1650296

**Published:** 2025-10-08

**Authors:** Yan Lian, Zhengkun Gan, Jieyu Sun, Jiawen Zhao, Qing Li, Xile Shen, Mingdong Xue, Junke Li, Jiaxin Luo, Xinyu Liu, Wuwen Feng, Guihua Jiang

**Affiliations:** ^1^ State Key Laboratory of Southwestern Chinese Medicine Resources, School of Pharmacy, Chengdu University of Traditional Chinese Medicine, Chengdu, China; ^2^ College of Ethnic Medicine, Chengdu University of Traditional Chinese Medicine, Chengdu, China

**Keywords:** migraine, Angelica dahurica radix, gut microbiota, metabolomics, gut-brain axis

## Abstract

**Background:**

Emerging evidence links gut-brain axis dysregulation to migraine pathogenesis. *Angelica dahurica* Radix (Baizhi) demonstrates clinical efficacy in migraine management, yet its mechanisms involving gut microbiota-metabolite crosstalk remain unelucidated.

**Methods:**

A nitroglycerin (NTG)-induced migraine rat model was established. Migraine behaviors were video-recorded. Enzyme-linked immunosorbent assay (ELISA) was used to quantify neuroinflammatory and neurotransmitter markers (5-HT, CGRP1, TNF-α, NO, PGE2, DA) in plasma and brain tissue. Intestinal barrier integrity was evaluated by immunohistochemical staining of tight junction proteins (Occludin/ZO-1) and hematoxylin-eosin (H&E) staining of colonic tissue. Gut microbiota composition was analyzed via 16S rRNA gene sequencing of fecal samples, and serum metabolomic profiles were characterized using ultra-high-performance liquid chromatography-quadrupole time-of-flight mass spectrometry (UPLC-Q-TOF MS). Multi-omics data were integrated to identify key therapeutic targets and pathways.

**Results:**

Baizhi alleviated migraine behaviors (head-scratching frequency reduced) and normalized neurochemical dysregulation (plasma and brain tissue 5-HT, CGRP1, TNF-α reduced vs. model). It restored intestinal barrier integrity via Occludin/ZO-1 upregulation and suppressed colonic inflammation. Gut microbiota analysis demonstrated that Baizhi increased the abundance of *Firmicutes* and beneficial genera, such as *Lactobacillus* and *Ruminococcus_gnavus_group,* while decreasing *Bacteroidetes*. Gut microbiota remodeling correlated with F/B ratio elevation and *Lactobacillus* enrichment. Key regulated pathways included tryptophan metabolism (5-HT synthesis) and mitochondrial-associated arginine-proline metabolism (NO regulation).

**Conclusion:**

Baizhi alleviates migraine through multi-target coordination: reinforcing gut barrier function, enriching anti-inflammatory *Lactobacillus*, and restoring neurotransmitter/neurovascular homeostasis through 2-AG-mediated CGRP1 inhibition. These findings bridge traditional herbology with modern neuromicrobiology, positioning BZ as a promising gut microbiota-modulating therapy for migraine.

## 1 Introduction

Migraine is a prevalent primary headache disorder, imposing a substantial global burden. According to the Global Burden of Disease 2019 study, in 2019, the global incidence of migraine increased to 87.6 million (95% UI: 76.6, 98.7), with a 40.1% increase compared to 1990 (Fan et al., 2023). Moreover, data from the American Migraine Prevalence and Prevention Study indicated that the prevalence of chronic migraine in the US population was nearly 1%, being highest among females, in mid - life, and in households with the lowest annual income (Buse et al., 2012). The pathogenesis of migraine involves multifactorial mechanisms, including neurovascular dysfunction, neurotransmitter imbalance (e.g., serotonin and calcitonin gene-related peptide) (Ashina et al., 2021), genetic predisposition, and ion channel abnormalities, which have not yet been fully elucidated (Goadsby et al., 2002). Both metabolite formulations and single-herb preparations in traditional Chinese medicine (TCM) have demonstrated distinct therapeutic advantages in migraine management (Wang et al., 2019). For instance, *Angelica Dahurica* Radix (Baizhi), a widely used TCM herb with a long clinical history, is recognized for its efficacy in dispelling wind-dampness, alleviating pain, and relieving nasal obstruction (Chinese Pharmacopoeia Commission. 2020). Baizhi has been widely used in traditional Chinese medicine for centuries. In traditional Chinese medicine classic works like *Compendium of Materia Medica*, BZ is recorded to “dispel wind and relieve pain, especially effective for headache due to wind - cold”,which lays a theoretical foundation for its historical application in headache treatment. Multiple studies (Liu et al., 2024; Chen et al., 2024; Lee et al., 2017; Yang et al., 2011) have reported its traditional applications in treating headache, including migraine, and its efficacy has also been demonstrated in some modern clinical practices.

Emerging pharmacological studies highlight Baizhi’s bioactive metabolites, particularly coumarins such as imperatorin and isoimperatorin, which regulate cerebrovascular tone by modulating calcium-dependent signaling pathways, thereby alleviating migraine symptoms induced by pathological vasoconstriction (Ni and Wang, 2018). Additionally, Baizhi’s volatile oils can penetrate the blood-brain barrier (BBB) to influence central neurotransmitter metabolism. For example, they stabilize serotonin (5-HT) levels, counteracting dysregulation associated with migraine pathogenesis. Baizhi also exhibits potent anti-inflammatory effects by suppressing pro-inflammatory mediators (e.g., interleukin-1*β* (IL-1β) and tumor necrosis factor-α (TNF-*α*)), thereby attenuating neuroinflammation and nociceptor sensitization (Tang et al., 2021). *In vitro* studies using neuroinflammatory cell models confirm that Baizhi extracts reduce inflammatory cytokine secretion and ameliorate cellular inflammatory states (Wei et al. (2016). *In vivo* experiments reveal that Baizhi administration reduces meningeal inflammatory cell infiltration and suppresses COX-2/iNOS expression via NF-κB signaling inhibition in NTG-induced migraine models (Chen et al., 2022). These advances have largely focused on a single metabolite (e.g., Imperatorin) and lack a systems biology perspective.

Recent advances in neurogastroenterology have underscored the gut-brain axis as a pivotal bidirectional communication network integrating gut microbiota, intestinal barrier integrity, immune responses, vagal signaling, and neuroendocrine pathways (Mayer et al., 2015). Microbial interventions (e.g., probiotics, fecal microbiota transplantation) have shown promise in mitigating neurobehavioral deficits in preclinical models, suggesting potential applications in migraine management (Bäckhed et al., 2004). This study employed ultra-high-performance liquid chromatography-quadrupole time-of-flight mass spectrometry (UHPLC-Q-TOF/MS) and 16S rRNA sequencing to analyze plasma metabolomic profiles and gut microbiota composition in a NTG-induced migraine rat model treated with Baizhi. By integrating multi-omics approaches, we aimed to elucidate Baizhi’s therapeutic mechanisms through its modulation of dysbiotic microbiota and host metabolic pathways, providing foundational insights for future translational research.

## 2 Materials and methods

### 2.1 Extraction procedure of BZ

Baizhi was sourced from Baoding, Hebei Province, authenticated as the dried root of *Angelica dahurica* (Fisch.ex Hoffm.) Benth. et Hook. f. by Professor Guihua Jiang (Chengdu University of Traditional Chinese Medicine). The species Angelica dahurica, the botanical drug investigated herein, is covered in the monograph of national pharmacopoeias (Chinese Pharmacopoeia Commission, 2020). After collection, the Baizhi was washed to remove soil and impurities, then dried in an oven at a set temperature of 50 °C until thoroughly dried. Voucher specimens deposited in the university’s herbarium (voucher No. BZ-2024-01, consistent with Sun et al., 2024).

200 g Baizhi was measured and combined with 10 times its volume of double-distilled water. The mixture was soaked for 1 h, followed by decoction for 30 min. After filtration, 8 times the amount of water was added to the filtrate and boiled again for 30 min. The two resulting fractions were combined and concentrated to a 0.5 g crude drug/mL concentration. The preparation, stored in a 4 °C refrigerator, yielded the BZ extract. Through this protocol, the dry extract rate of the BZ extract was calculated to be 10.77%.

### 2.2 Qualitative and quantitative analysis of BZ

#### 2.2.1 Qualitative analysis by UPLC

Chromatographic methods for the qualitative analysis of BZ were conducted as previously reported (Zhe et al., 2025). Mass spectrometric methods for the qualitative analysis of BZ were performed using Ultra Performance Liquid Chromatography-Quadrupole-Orbitrap High Resolution Mass Spectrometry (UPLC-Q-Exactive Orbitrap MS), Zeno TOF 7600, AB Sciex).

The Waters CORTECS T3 UPLC C18 column (1.6 μm, 2.1 × 150 mm) was employed with a column temperature of 35 °C, flow rate of 0.25 mL/min, injection volume of 3 μL, and detection wavelength at 305 nm. The mobile phase consisted of 0.1% acetic acid in water-acetonitrile, with a gradient elution program: 0–5 min (90%→55%,10%→45%); 5–12 min (55%→40%,45%→60%); 12–16 min (40%→36%,60%→64%); 16–26 min (36%→10%,64%→90%); 26–28 min (10%→90%,90%→10%); 28–30 min maintaining the initial ratio (90%,10%) for equilibrium.

The TOF MS-IDA-MS/MS mode was employed with an ESI ion source (positive/negative ion scanning). The first-stage mass spectrum ranged from m/z 100 to 1,500, while the second-stage mass spectrum spanned m/z 50 to 1,500. Key parameters included: ion source temperature at 550 °C, curtain gas pressure at 35 psi, nebulizer and auxiliary gas pressure both set at 55 psi; electrospray voltage between 5500 V and -4500 V, deionization voltage at ±80 V; collision energy: 10 V for TOF-MS mode ±and 45 ± 20 V to-45 ± 15 V for IDA-MS/MS mode; Zeno threshold of 10,000,000 collisions per second (cps). Signal acquisition was performed using SCIEX OS 3.0 software.

#### 2.2.2 Quantitative analysis by HPLC

Chromatographic analysis was conducted using an Agilent 1,260 Infinity Ⅱ high-performance liquid chromatograph (Agilent Technologies) with a COSMOSIL C18-MS-II column (100 × 2.1 mm, 1.8 μm) maintained at 25 °C (Michael H,Banaz J,Mona T A, et al., 2022). A binary mobile phase system consisting of (A) 0.1% formic acid in water and (B) acetonitrile was employed with gradient elution as follows: 0 min (85.0% A, 15.0% B); 5.50 min (72.0% A, 28.0% B); 7.00 min (60.0% A, 40.0% B); 9.50 min (60.0% A, 40.0% B); 16.00 min (50.0% A, 50.0% B); 18.00 min (35.0% A, 65.0% B); 20.00 min (85.0% A, 15.0% B). The flow rate was 0.35 mL/min, and detection was performed at 300 nm. A mixed standard solution containing Byakangelicol, Oxypeucedanin hydrate, and Bergapten with mass concentrations of 0.144 mg/mL, 0.0424 mg/mL, and 0.014 mg/mL, respectively, was prepared by dissolving in methanol. The injection volume was 2 μL.

### 2.3 Animal experiments

The study protocol received ethical approval (2019-08) from the Ethics Committee of Chengdu University of Traditional Chinese Medicine, with all procedures conducted in strict accordance with established animal welfare standards. Specific-pathogen-free (SPF)-grade adult male Wistar rats (190 ± 20 g) were obtained from Chengdu Dasuo Experimental Animal Co., Ltd (License No.: SCXK (Sichuan) 2020-0030), and were maintained at 22 °C ± 2 °C with a 12 h light/dark cycle with ad-libitum access to food and water in the animal facility. After 1 week acclimation period, the rats were randomly assigned to four groups (randomized using a computer-generated sequence): Control group (Normal), Model group (Model), BZ group (BZ) and positive drug group (Sumaputan succinate injection, SS). The Normal and Model groups received intragastric administration of sterile water (10 mL/kg) for 14 days, while the BZ group was administered BZ (7.2 g crude drug/kg, corresponds to 0.78 g extract/kg) orally for 14 consecutive days. Previous investigations into dose - response effects (Chen et al., 2023; Sun et al., 2024; Lei et al., 2021). offered crucial guidance for determining the dosage employed in this study. The Normal group received no treatment, while the Model group, SS group, and BZ group were subcutaneously injected with NTG to induce migraine - like symptoms (10 mg/kg, Beijing Yimin Pharmaceutical Co., Ltd., Beijing, China) every other day for 9 days (5 times in total) (Hao et al., 2022).

### 2.4 Biochemical measurements in plasma and brain

The concentrations of factors 5-HT, NO, CGRP1, ET-1, PGE2, TNF-*α*, and DA in both plasma and brain tissue of the rats were quantified using commercial enzyme-linked immunosorbent assay (ELISA) kits (Elabscience, Wuhan, China; Cat. No.: 5-HT, E-EL-0033; CGRP1, E-EL-R0135; TNF-α, E-EL-R2856; ET-1, E-EL-R1458; PGE2, E-EL-0034; DA, E-EL-0046; NO, E-BC-K035-M). All assays were conducted in strict adherence to the manufacturer’s protocols. Some results of ELISA were expressed as the ratio of the target substance content (ng) to the total protein amount (mg) (ng/mg).

### 2.5 Histopathological examination

To evaluate intestinal barrier integrity, colonic tissues were fixed in 4% paraformaldehyde for 24 h, embedded in paraffin, and sectioned into 5 μm slices. Hematoxylin and eosin (H&E) staining was performed following standard protocols (Lanza et al., 2021). Histopathological changes (e.g., inflammatory cell infiltration, mucosal epithelial necrosis, crypt degeneration, and lamina propria edema) were examined under a light microscope (Olympus CX23, Japan) at ×200 magnification. Three randomly selected fields per sample were analyzed using ImageJ software (v1.53, NIH, USA) to quantify pathological features.

### 2.6 Immunohistochemical staining

Tight junction proteins Occludin and ZO-1 were assessed to determine intestinal barrier function. Deparaffinized sections underwent antigen retrieval in citrate buffer (pH 6.0) at 95 °C for 20 min. After blocking with 5% bovine serum albumin (BSA), sections were incubated overnight at 4 °C with primary antibodies: anti-Occludin (1:200, Abcam, Cat# ab216327) and anti-ZO-1 (1:200, Abcam, Cat# ab276131). Subsequently, horseradish peroxidase (HRP)-conjugated secondary antibodies (1:5000, Servicebio, China) were applied for 1 h at room temperature. Diaminobenzidine (DAB) was used for chromogenic detection, and nuclei were counterstained with hematoxylin. Protein expression levels were quantified as integrated optical density (AOD) using ImageJ software.

### 2.7 16S rRNA-based microbial community analysis

Microbial DNA extraction from feces samples was performed using the E.Z. N.A.^®^ soil DNA kit (Omega Bio-tek, Norcross, GA, U.S.) following the manufacturer’s protocol. The V3–V4 region of the bacterial 16S rRNA gene was amplified using a thermal cycler PCR system (GeneAmp 9,700, ABI, USA). The paired-end sequence data from MiSeq sequencing were merged based on their overlap. Following quality control and filtering, operational taxonomic units (OTUs) with 97% similarity were clustered using USEARCH (version 7.0 http://drive5.com/USEARCH/), and chimeric sequences were subsequently removed.

The UPARSE software was employed for operational taxonomic unit (OTU) clustering of the sequences based on a 97% similarity threshold (Chen et al., 2025). The Rhonin Biosciences cloud platform (http://www.biomediv.cn/login.html) was utilized for analysis. Principal coordinates analysis (PCoA) and Venn diagrams were used to evaluate the differences in bacterial community structure across the samples. Linear discriminant analysis coupled with effect size (LEfSe) was conducted to identify the dominant microbiota from phylum to genus, according to a standard LDA score of >3, *p* < 0.05 in different groups (Tian et al., 2023).

### 2.8 Serum metabolomics analysis

Serum samples from each group were collected in metabolic cages and stored in a −80 °C refrigerator until analysis. Four times the volume of cold methanol was added to the precisely weighed samples and homogenized thoroughly. The mixture was then subjected to ultrasonic treatment in an ice bath for 20 min. Subsequently, the supernatant was centrifuged at 13,000 rpm for 10 min at 4 °C. Metabolomic analysis was performed using a ThermoFisher UHPLC(ThermoFisher Scientific, MA, USA) system coupled with an Orbitrap mass spectrometer. For both Electrospray ion source (ESI) positive and negative modes, the mobile phases consisted of A (0.1% formic acid in water) and B (0.1% formic acid in acetonitrile), with a flow rate of 0.3 mL/min.

ESI was utilized for positive ion mode detection. The operating parameters were as follows: spray voltage at 3.2 kV, ion source temperature at 350 °C, sheath gas flow rate at 35 arb, auxiliary gas flow rate at 10 arb, and ion transport tube temperature at 320 °C. The instrument employed a full scan/data-dependent secondary scan (Full MS/dd-MS2) mode with a first-level resolution of 70,000 and a second-level resolution of 17,500. The scan range was set from m/z 100 to 1,500, with a collision energy gradient of 20, 40, and 60 eV.

Non-targeted serum metabolomics raw data were imported into the Compound Discoverer 3.3 software. Through its wizard setup and method template, a process for identifying unknown metabolites was established. Peak alignment and extraction of the raw data were performed, fitting the extracted molecular ion chromatography peaks and isotopic peaks to possible molecular formulas. The secondary fragmentation spectra were matched with mzCloud, mzvault network database, and the local TCM composition database OTCML. Filter parameters for the matching results were set as follows: peak area threshold of 80,000, primary and secondary mass deviations of 5 ppm, and a match degree score above 80. Statistical analysis was then conducted. Metaboanalyst 6.0 software (http://www.metaboanalyst.ca/) and Metware Cloud (https://cloud.metware.cn) were used to perform principal components analysis (PCA), partial least squares discriminant analysis (PLS-DA), heatmap analysis, and metabolic enrichment and pathway analysis. The selection of significantly different metabolites was determined based on the variable importance in the projection (VIP) obtained using the PLS-DA model and the p-value of the Student’s t-test. Differential metabolites were screened via the Metware Cloud using criteria: VIP ≥1.0, *p* < 0.05, and False Discovery Rate(FDR) ≤ 0.2. These metabolites were imported into MetaboAnalyst 6.0 for pathway analysis. The analysis compared three groups (Normal vs. Model vs. BZ), prioritizing Model vs. BZ to dissect BZ - mediated metabolic rescue, while Normal vs. Model data defined baseline migraine - associated dysmetabolism.

### 2.9 Correlation analysis between metabolites and intestinal flora

The Spearman correlation coefficient was employed to illustrate the relationship between the parameters (linear correlation). This coefficient consistently ranges between −1 and +1, with values closer to either extreme indicating a stronger linear relationship. In this study, Spearman correlation coefficients *r* > 0.6 and r < −0.6 were designated to signify significant positive and negative correlations, respectively.

### 2.10 Statistical analysis

All data were presented as mean ± standard error of the mean (SEM). Statistical analyses were performed using GraphPad Prism 10.4 software. Parametric and non-parametric data were compared using the independent t-test and Mann-Whitney U test, respectively (2 groups). For comparisons involving three or more groups, one-way analysis of variance (ANOVA) was first conducted to evaluate overall differences among groups. Subsequent pairwise comparisons were performed using Tukey’s multiple comparison test to identify specific group differences. A value of *p* < 0.05 was considered statistically significant.

## 3 Results

### 3.1 Chemical constitution in the aqueous extract of BZ

**FIGURE 1:**
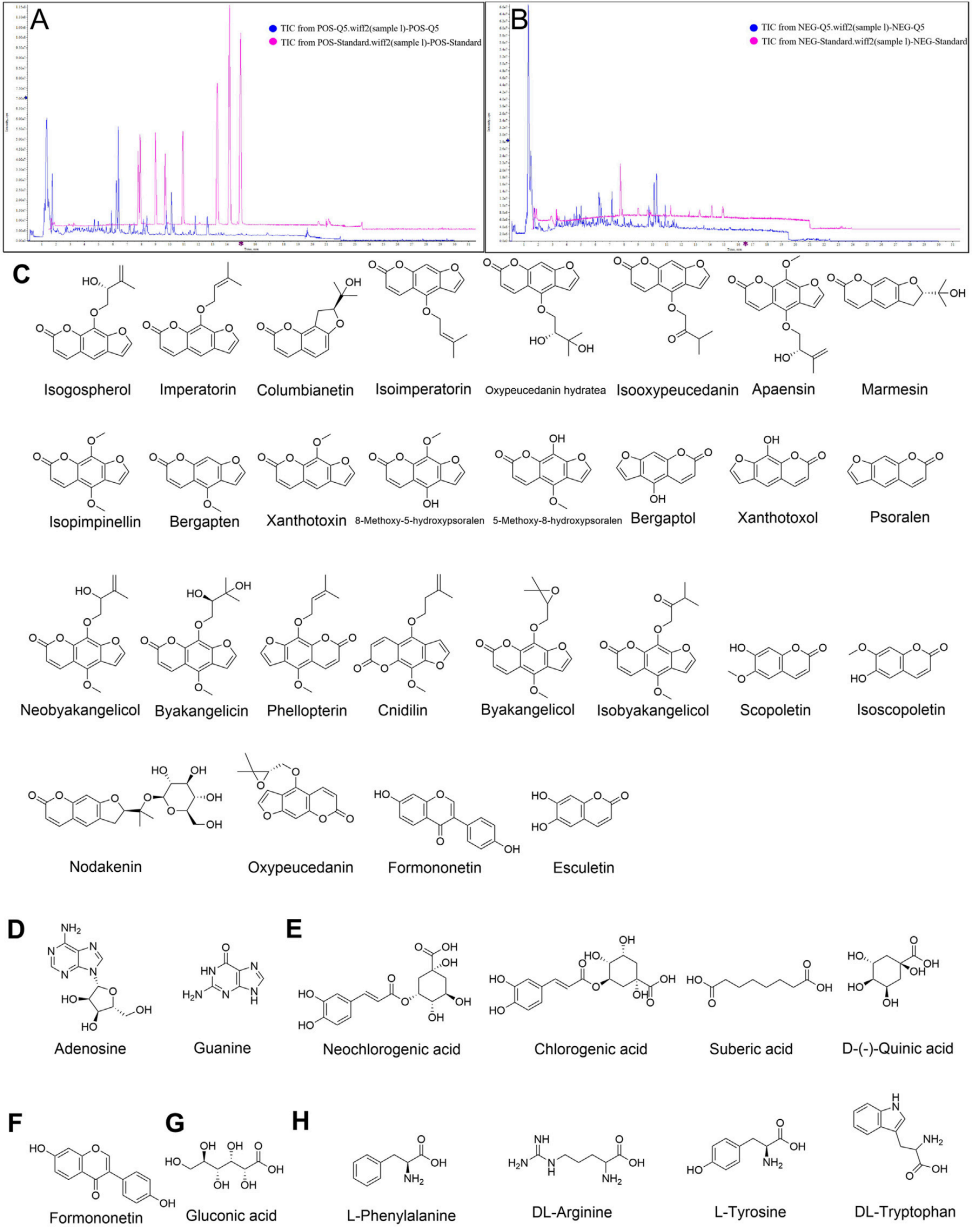
Qualitative analysis of BZ and metabolites identified. Total ion chromatogram of BZ in **(A)** Positive ion mode and **(B)** Negative ion mode. **(C)** coumarins, **(D)** nucleosides, **(E)** organic acids, **(F)** flavonoids, **(G)** sugars and **(H)** amino acids in BZ were identified by UPLC-Q-Exactive Orbitrap MS.

As shown in Figure 1A; Table 1, 40 chemical constituents were detected by UPLC-Q-Exactive Orbitrap MS, whose structural formulas were displayed in Figures 1C–H. The contents of three target metabolites in the Baizhi extract were determined as follows: Oxypeucedanin hydrate was 0.2193 mg/g, Byakangelicol was 0.2101 mg/g, and Bergapten was 0.0152 mg/g (n = 3). For detailed information, please refer to Supplementary Material 1.

**TABLE 1:** Chemical metabolites of BZ identified by UPLC-Q-Exactive Orbitrap MS.

NO	RT/min	Name	Formula	Ion	Measured (M/Z)	Error (ppm)	MS/MS fragment ions	Classifcation
1	1.214	DL-Arginine	C_6_H_14_N_4_O_2_	[M-H]^-^	173.1043	−0.4	58.0416、59.0168、83.0623、112.0864、131.0834、136.9353	H
2	1.302	D-(−)-Quinic acid	C_7_H_12_O_6_	[M-H]^-^	191.0557	−2.2	57.0355、67.0205、85.0306、87.0105、93.0352、111.0100	D
3	1.306	Gluconic acid	C_6_H_12_O_7_	[M-H]^-^	195.0506	−2.2	59.0151、75.0108、72.9929、87.0086、99.0085	G
4	1.445	Neochlorogenic acid	C_16_H_18_O_9_	[M-H]^-^	353.0874	−1.0	85.0302、111.0093、135.0465、179.0356、191.0573	E
5	1.445	Chlorogenic acid	C_16_H_18_O_9_	[M-H]^-^	353.0874	−1.0	85.0302、111.0093、135.0465、179.0356、191.0573	E
6	1.718	Adenosine	C_10_H_13_N_5_O_4_	[M + H]^+^	268.1038	−0.9	57.0333、94.0402、119.0367、136.0700、268.1060	D
7	1.719	L-Tyrosine	C_9_H_11_NO_3_	[M-H]^-^	180.0657	−5.3	72.0128、93.0353、94.0431、119.0516、134.0685、136.0875	H
8	1.721	Guanine	C_5_H_5_N_5_O	[M + H]^+^	152.0562	−3.1	55.0288、109.0517、110.0352、135.0294、152.0584、153.0368	D
9	2.762	DL-Tryptophan	C_11_H_12_N_2_O_2_	[M-H]^-^	203.0822	−1.9	74.0258、81.8625、116.0503、130.0648、140.0494、142.0663	H
10	3.252	7-Hydroxycoumarine	C_9_H_6_O_3_	[M + H]^+^	163.0382	−4.7	60.9861、63.0230、89.0384、117.0333、135.0439、145.0179、163.0384	C
11	3.837	Esculetin	C_9_H_6_O_4_	[M-H]^-^	177.0187	−3.6	51.0233、77.0399、89.0395、105.0353、133.0298、177.0187	C
12	3.849	L-Phenylalanine	C_9_H_11_NO_2_	[M + H]^+^	166.0858	−2.9	69.0333、80.0491、87.0439、118.0645、136.0757、148.0749、166.0863	H
13	4.512	Suberic acid	C_8_H_14_O_4_	[M-H]^-^	173.0811	−4.9	57.0329、59.0130、83.0494、109.0649、111.0857、136.9351	E
14	4.685	Columbianetin	C_14_H_14_O_4_	[M + H]^+^	247.0960	−2.0	91.0545、147.0436、175.0406、176.0464、229.0880、247.1021	C
15	4.685	Marmesin	C_14_H_14_O_4_	[M + H]^+^	247.0960	−2.0	91.0545、147.0436、175.0406、176.0464、229.0880、247.1021	C
16	4.689	Nodakenin	C_20_H_24_O_9_	[M + H]^+^	409.1485	−1.9	85.0284、159.0443、175.0391、187.0388、229.0852、247.0989	C
17	4.881	Scopoletin	C_10_H_8_O_4_	[M + H]^+^	193.0491	−2.0	94.0409、133.0278、137.0603、150.0307、178.0254、193.0497	C
18	4.881	Isoscopoletin	C_10_ H_8_O_4_	[M + H]^+^	193.0491	−2.0	193.0497、133.0278、178.0254、137.0603、150.0307	C
19	5.891	Formononetin	C_16_H_12_O_4_	[M + H]^+^	269.0802	−2.3	89.0389、145.0286、173.0243、201.0196、203.0338、269.0815	F
20	6.246	Bergaptol	C_11_H_6_O_4_	[M-H]^-^	201.0195	0.7	65.0044、89.0404、101.0407、117.0426、145.0323、201.0224	C
21	6.249	Oxypeucedanin hydratea	C_16_H_16_O_6_	[M + H]^+^	305.1017	−0.9	57.0717、59.0551、131.0528、147.0506、203.0396、305.1036	C
22	6.384	5-Methoxy-8-hydroxypsoralen	C_12_H_8_O_5_	[M + H]^+^	233.0438	−2.8	78.0489、134.0369、173.0237、190.0265、218.0289、233.0502	C
23	6.384	8-Methoxy-5-hydroxypsoralen	C_12_H_8_O_5_	[M + H]^+^	233.0438	−2.8	78.0489、134.0369、173.0237、190.0265、218.0289、233.0502	C
24	6.385	Byakangelicin	C_17_H_18_O_7_	[M + H]^+^	335.1121	−1.3	67.0547、85.0649、175.0396、203.0337、231.0323、233.0453	C
25	7.208	Psoralen	C_11_H_6_O_3_	[M + H]^+^	187.0383	−3.6	77.0426、103.0542、115.0563、131.0530、143.0507、187.0436	C
26	7.497	Xanthotoxin	C_12_H_8_O_4_	[M + H]^+^	217.0492	−1.3	90.0486、118.0426、146.0370、174.0347、202.0294、217.0560	C
27	8.160	Bergapten	C_12_H_8_O_4_	[M + H]^+^	217.0493	−1.3	90.0527、118.0465、146.0383、174.0381、202.0329、217.0547	C
28	8.183	Isopimpinellin	C_13_H_10_O_5_	[M + H]^+^	247.0596	−1.8	95.0126、161.0239、189.0188、217.0188、232.0378、247.0633	C
29	8.336	Isogospherol	C_16_H_14_O_5_	[M + H]^+^	287.0911	−0.9	91.0587、131.0501、147.0477、159.0452、203.0413、287.0934	C
30	8.336	Isooxypeucedanin	C_16_H_14_O_5_	[M + H]^+^	287.0911	−0.9	91.0587、131.0501、147.0477、159.0452、203.0413、287.0934	C
31	8.336	Oxypeucedanin	C_16_H_14_O_5_	[M + H]^+^	287.0911	−0.9	91.0587、131.0501、147.0477、159.0452、203.0413、287.0934	C
32	9.687	Neobyakangelicol	C_17_H_16_O_6_	[M + H]^+^	317.1015	−1.4	67.0542、175.0393、203.0335、218.0207、231.0293、233.0459	C
33	9.687	Apaensin	C_17_H_16_O_6_	[M + H]^+^	317.1015	−1.4	67.0542、175.0393、203.0335、218.0207、231.0293、233.0459	C
34	9.687	Byakangelicol	C_17_H_16_O_6_	[M + H]^+^	317.1015	−1.4	67.0542、175.0393、203.0335、218.0207、231.0293、233.0459	C
35	9.687	Isobyakangelicol	C_17_H_16_O_6_	[M + H]^+^	317.1015	−1.4	67.0542、175.0393、203.0335、218.0207、231.0293、233.0459	C
36	11.794	Imperatorin	C_16_H_14_O_4_	[M + H]^+^	271.0958	−2.4	69.0713、91.0556、131.0496、147.0479、175.0396、203.0402	C
37	11.795	Xanthotoxol	C_11_H_6_O_4_	[M + H]^+^	203.0334	−2.3	65.0419、91.0581、131.0508、147.0506、175.0413、203.0404	C
38	12.697	Phellopterin	C_17_H_16_O_5_	[M + H]^+^	301.1072	0.6	69.0714、134.0371、217.0138、218.0268、230.0215、233.0504	C
39	12.697	Cnidilin	C_17_H_16_O_5_	[M + H]^+^	301.1072	0.6	69.0714、134.0371、217.0138、218.0268、230.0215、233.0504	C
40	13.479	Isoimperatorin	C_16_H_14_O_4_	[M + H]^+^	271.0957	−2.7	69.0699、91.0545、131.0492、147.0443、159.0442、203.0361	C

(C)coumarins, (D) nucleosides, (E) organic acids, (F) flavonoids, (G) sugars and (H) amino acids

### 3.2 BZ ameliorated NTG-induced migraine symptoms in rats

**FIGURE 2:**
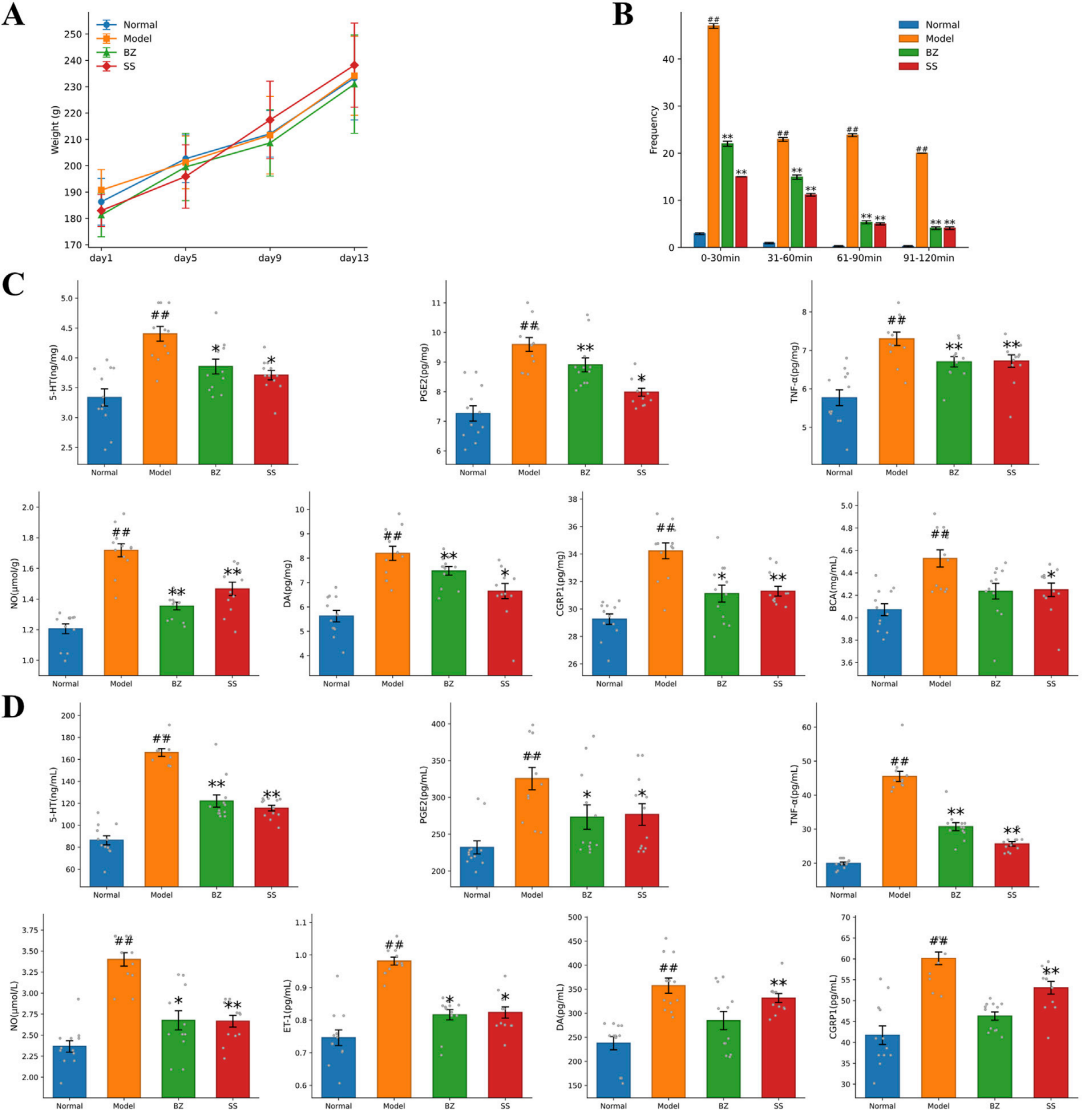
BZ improved migraine-related biochemical indexes in rats. **(A)** Body weight of rats. **(B)** Frequency of head scratching instances in each group. **(C)** Biochemical indexes of brain tissue. **(D)** Plasma biochemical indexes. The values are expressed as the Mean ± Standard deviation (n = 12 rats/group). Differences were evaluated by t tests (^#^ *P* < 0.05 vs. Normal group, ^##^ *P* < 0.01 vs. Normal group, ^*^ *P* < 0.05 vs. Model group, ^**^ *P* < 0.01 vs. Model group).

Throughout the experimental period (Figure 2A), the Model group exhibited a slight decrease in body weight, while the groups of BZ and SS treatment groups demonstrated an increasing trend similar to the Normal group. The frequency of head scratching increased after NTG injection, the model group showed a significantly increased total average head-scratching frequency (113 times within 120 min) compared with the normal group, while BZ group reduced the total average head-scratching frequency by 61.2% vs. Model group (*p* < 0.01) (Figure 2B). ELISA assay results revealed significantly elevated levels of 5-HT, NO, CGRP1, TNF-*α*, PGE2, and DA in the Model group compared to the Normal group. Notably, these levels were significantly reduced in the BZ group relative to the Model group. In brain tissue, BZ group reduced the levels of CGRP1 by 9.6% (*p* < 0.05), DA by 7.5% (*p* > 0.05), 5-HT by 11.5% (*p* < 0.05), TNF-α by 9.7% (*p* < 0.05), PGE_2_ by 7.2% (*p* > 0.05), and NO by 20.5% (*p* < 0.01) compared with the Model group. In plasma, BZ group reduced the levels of CGRP1 by 20.5% (*p* < 0.01), DA by 20.3% (*p* < 0.05), 5-HT by 24.2% (*p* < 0.01), TNF-α by 31.2% (*p* < 0.01), NO by 20.2% (*p* < 0.01), and ET-1 by 17.6% (*p* < 0.05) compared with the Model group. Collectively, these findings suggest that BZ administration alleviated NTG-induced migraine-like symptoms.

### 3.3 BZ enhances intestinal barrier in NTG-induced rats

**FIGURE 3:**
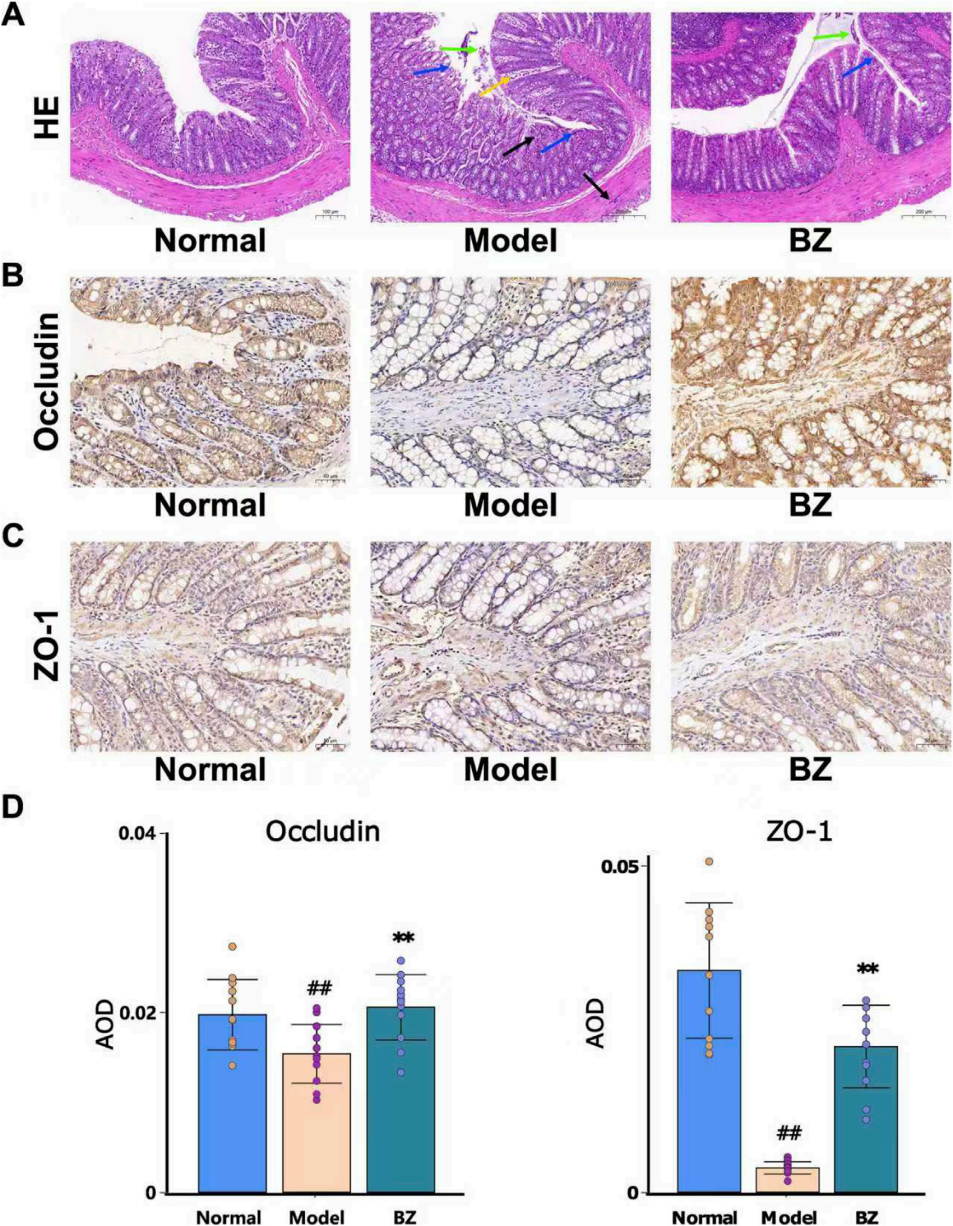
BZ relieved disrupted intestinal barrier function in NTG-induced migraine rats. **(A)** Representative HE staining images (20.0×). **(B)** Representative IHC of Occludin. **(C)** Representative IHC of ZO-1. **(D)** The expression level of Occludin and ZO-1 was analyzed by Image J. The values are expressed as the Mean ± Standard deviation (n = 12 rats/group). Differences were evaluated by t tests (^##^ *P* < 0.01 vs. Normal group, ^**^ *P* < 0.01 vs. Model group).

Intestinal barrier dysfunction plays a significant role in the pathogenesis of migraine (Lanza et al., 2021). We hypothesize that the efficacy of BZ in NTG-induced migraine may be closely associated with enhanced intestinal barrier function. Because Occludin/ZO-1 are pivotal for intestinal tight - junctions, we measured their protein levels to assess intestinal barrier integrity in migraine mice and the impact of BZ treatment. Histological changes in the colonic tissue were evaluated using H&E-stained tissue sections (Figure 3A). As shown by the arrow - marked areas in Figure 3A, the Model group presented severe damage: black arrows marked disrupted mucosal layers, blue arrows showed glandular distortion, yellow arrows indicated massive inflammatory cell infiltration, and green arrows denoted epithelial cell shedding. In the BZ group, distinct improvements appeared. As indicated by arrow - marked areas, yellow arrows showed a significant reduction in inflammatory cells compared to the Model group, black arrows revealed restored mucosal integrity, blue arrows showed more regular glands, and green arrows indicated reduced epithelial shedding. Overall, BZ treatment significantly alleviated colonic tissue damage.

Subsequently, immunohistochemistry was employed to explore the expression of migraine in relation to Occludin and ZO-1. As depicted in Figures 3B–D, in nitroglycerin-induced migraine model rats, the protein expression levels of Occludin and ZO-1 were significantly elevated after BZ treatment. These results indicate that BZ restores the impaired intestinal barrier function in NTG - induced migraine rats.

### 3.4 BZ modulated the gut microbiota composition in NTG-induced rats

**FIGURE 4:**
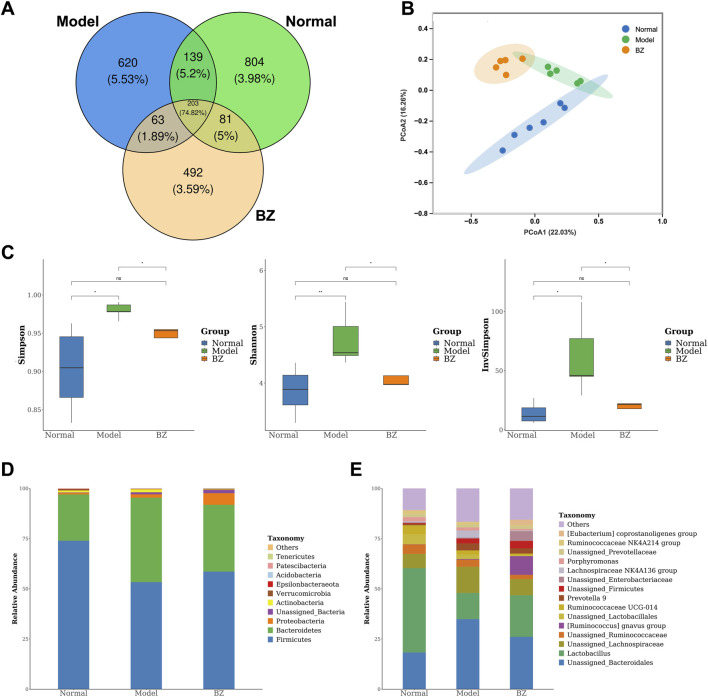
BZ shifted the overall gut microbiota composition in migraine rats. **(A)** Venn diagram showing the number of bacteria species in each group. **(B)** PCA diagram showing the overall structure of the gut microbiota of three groups (Normal group n = 6 rats, Model group n = 5 rats, BZ group n = 5 rats). **(C)** Alpha diversity of gut microbiota. **(D)** Bacterial taxa of gut microbiota at the phylum level. **(E)** Bacterial taxa of gut microbiota at the genus level. Differences were evaluated by t tests (^*^ *P* < 0.05, and ns represents no significant difference).

In order to further confirm whether the anti-migraine effect of BZ is dependent on the presence of the gut microbiota, we treated migraine rats with BZ. On the 15th day, we measured the composition of gut microbiota. Venn analysis revealed substantial microbial community divergence, with only 203 core OTUs shared among Normal, Model, and BZ groups (Figure 4A). The BZ group exhibited 492 unique OTUs *versus* 804 in Normal and 620 in Model groups, indicating treatment-specific microbiota reorganization. PCoA analysis (PC1: 22.03%, PC2: 16.26%) confirmed BZ’s capacity to shift microbial *β*-diversity toward normative clustering (Figure 4B). *α*-Diversity metrics demonstrated migraine-associated dysregulation: Model group showed elevated Shannon index and InvSimpson index, suggesting pathological hyperdiversity. BZ treatment normalized these indices, restoring microbial community equilibrium (Figure 4C). Microbiota analysis at phylum and genus levels (Figures 4D,E) identified distinct dysbiosis in migraine rats. At the phylum level, migraine rats had reduced Firmicutes and increased Bacteroidetes, lowering the *Firmicutes/Bacteroidetes* (F/B) ratio vs. Normal; BZ reversed this by elevating Firmicutes relative abundance and suppressing Bacteroidetes (Figure 4D). The F/B ratio is a key gut microbiota balance indicator. Previous studies (An et al., 2023; Spase et al., 2020; Koliada et al., 2017) note that an abnormal F/B increase links to intestinal dysbiosis and disease pathogenesis—e.g., it may drive ulcerative colitis progression or contribute to obesity (Spase et al., 2020). In our study, the Model group had a higher F/B ratio than the Normal group (indicating migraine-induced gut dysbiosis), while BZ reduced the F/B ratio, suggesting it restores microbiota balance and may aid its anti-migraine effect. At the genus level (Figure 4E), several genera showed significant differences among the groups. In the Model group, the relative abundances of genus *Lactobacillus* and genus *Unassigned_Bacteroidales* were significantly increased compared to the Normal group, while in the BZ group, the relative abundances of these genera were decreased, approaching the levels of the Normal group. These changes suggest that BZ treatment may regulate the composition of gut microbiota at the genus level.

**FIGURE 5:**
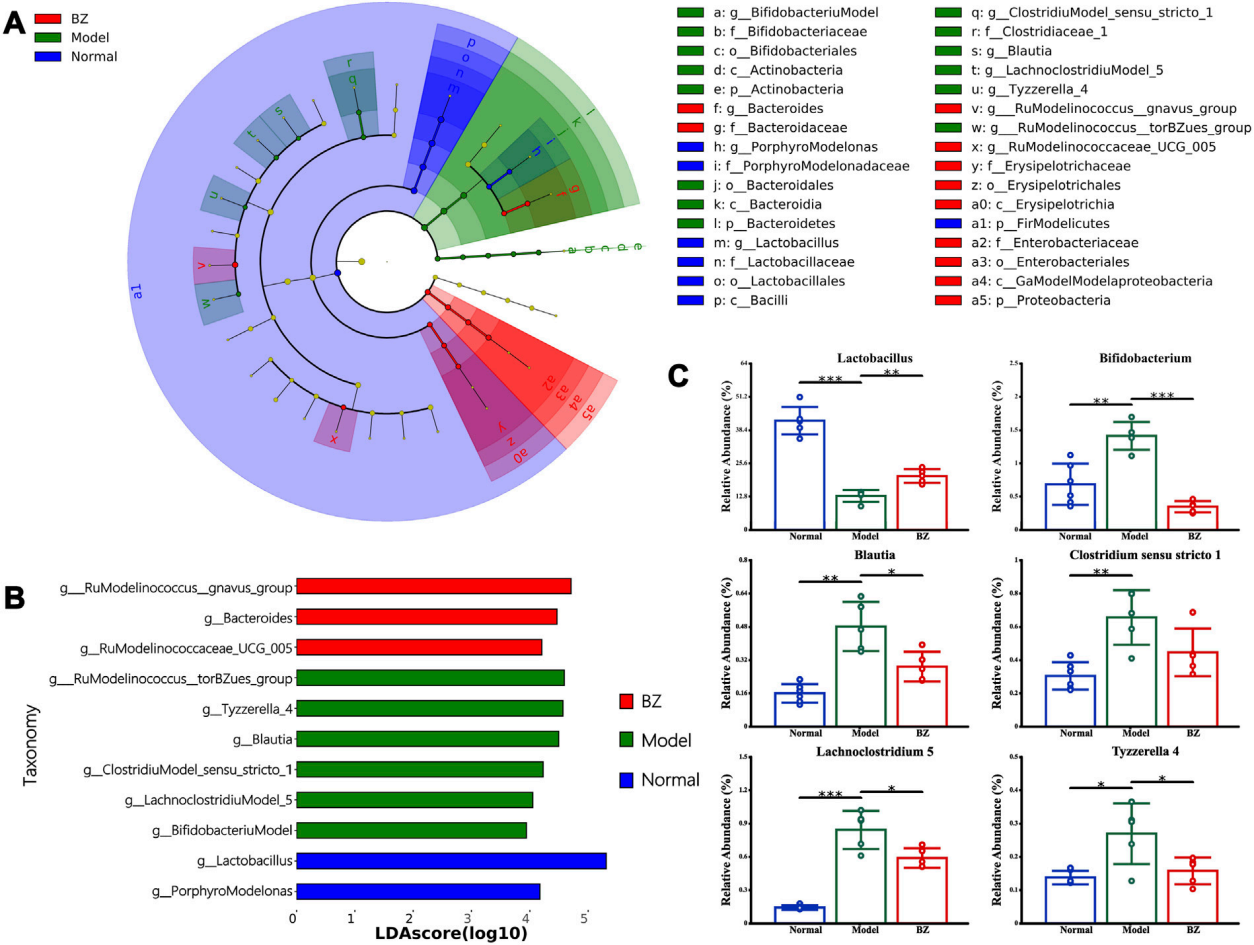
LEfSe analysis of the different gut microbiota in Normal, Model, and BZ groups. **(A)** Taxonomic cladogram gained from LEfSe analysis. **(B)** The LDA values were higher than 3. **(C)** Relative abundance of the key discriminative intestinal microbiota at genus level. The values are expressed as the Mean ± Standard deviation (Normal group n = 6 rats, Model group n = 5 rats, BZ group n = 5 rats). Differences were evaluated by t tests (^*^ *P* < 0.05, ^**^ *P* < 0.01, and ^***^ *P* < 0.001).

LEfSe analysis (LDA >3.0, *p* < 0.05) identified 32 discriminatory taxa across groups (Figure 5A). The Normal group was dominated by *Lactobacillus*, reflecting gut homeostasis, while the Model group exhibited enrichment of pro-inflammatory taxa including *[Ruminococcus] torques group* and *Bacteroides*. Conversely, the BZ group demonstrated a unique microbiota profile characterized by Erysipelotrichaceae and *Enterobacteriales*, alongside significant suppression of migraine-associated *[Ruminococcus] gnavus group*. Notably, bar plots in Figure 5C display the relative abundance of key taxa confirmed BZ-mediated restoration of beneficial *Lactobacillus* abundance, correlating with 5-HT recovery, while pathogenic *Porphyromonas* decreased, aligning with *Z O -1* upregulation.

### 3.5 BZ regulated the serum metabolomics in NTG-induced rats

**FIGURE 6:**
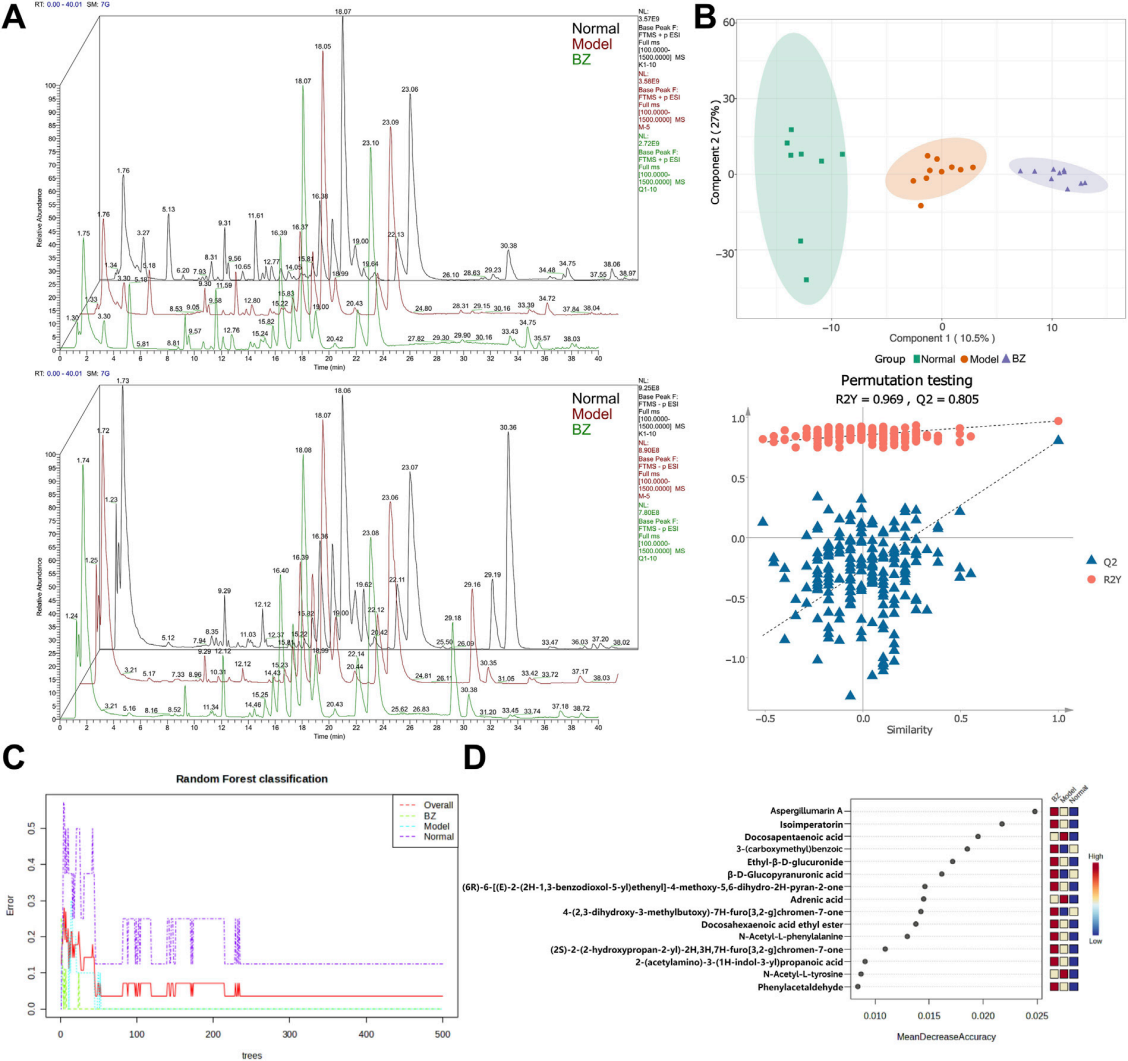
BZ treatment altered serum metabolite profile in migraine rats. **(A)** Diagram of the positive and negative ion flow of the three metabolic groups (Normal group n = 8 rats, Model group n = 10 rats, BZ group n = 9 rats). **(B)** PLS-DA analysis of the metabolites from the three groups. **(C, D)** The random forest classification and most relevant top 15 substances.

Non-targeted metabolomics was employed to investigate the changes in non-volatile metabolites in serum. Figure 6A reveals significant alterations in numerous metabolites compared to the Normal group, indicating that the establishment of the migraine model resulted in substantial changes in metabolite profiles in rats. Differences and similarities among the Normal, Model, and BZ groups were evaluated using PLS-DA (Figure 6B). The analysis revealed a significant separation between the Normal and Model groups. Furthermore, abundance analysis and random forest analysis were utilized to identify the top 15 most relevant substances (Figures 6C,D), which verified the similarities and differences among the Normal, Model, and BZ group (Xing et al., 2024).

**FIGURE 7:**
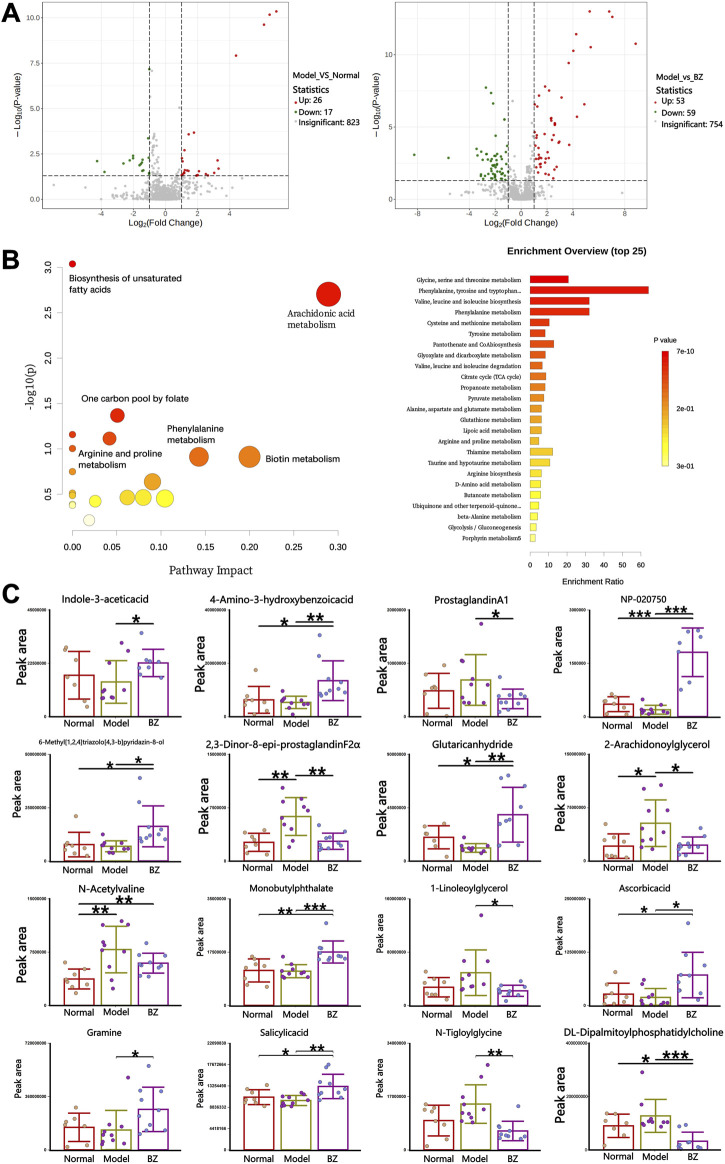
Specific serum metabolites modulated by BZ. **(A)** Volcano plot of the serum metabolites in each group. **(B)** Pathway analysis of the serum metabolites. **(C)** The content of each metabolite varies in different groups. The values are expressed as the Mean ± Standard deviation (Normal group n = 8 rats, Model group n = 10 rats, BZ group n = 9 rats). Differences were evaluated by t tests (^*^ *P* < 0.05, ^**^ *P* < 0.01, and ^***^ *P* < 0.001).

The volcano map in Figure 7A confirmed the similarities and differences between the Normal group and the Model group, as well as between the Model group and BZ group. Further analysis revealed that, compared to the Normal group, the relative peak areas of multiple metabolites in the Model group were significantly altered. Moreover, metabolomics pathway analysis of the inter-group differences demonstrated distinct enrichment patterns. Pathways such as arachidonic acid metabolism exhibited a high -log10(p) value and relatively high pathway impact, suggesting a significant association with the observed metabolic changes. Other pathways, including one carbon pool by folate and phenylalanine metabolism, also displayed varying degrees of significance and impact (Figure 7B). Following BZ treatment, the relative peak areas of numerous metabolites, including 4-Amino-3-hydroxybenzoic acid and ProstaglandinA1, showed a trend of reverting towards the levels observed in the Normal group. Among these, 2-AG associated with CGRP1 inhibition, and eight-epi-PGF2*α* associated with decreased NF-κB phosphorylation. This suggests that these metabolites may play crucial roles in the response to BZ treatment and the underlying biological processes among the different groups. Metabolomic pathway analysis identified significant enrichment in arachidonic acid metabolism, which is linked to neuroinflammatory responses in migraine.

### 3.6 Correlation analysis among gut microbiota, metabolites, and migraine-related indices

**FIGURE 8:**
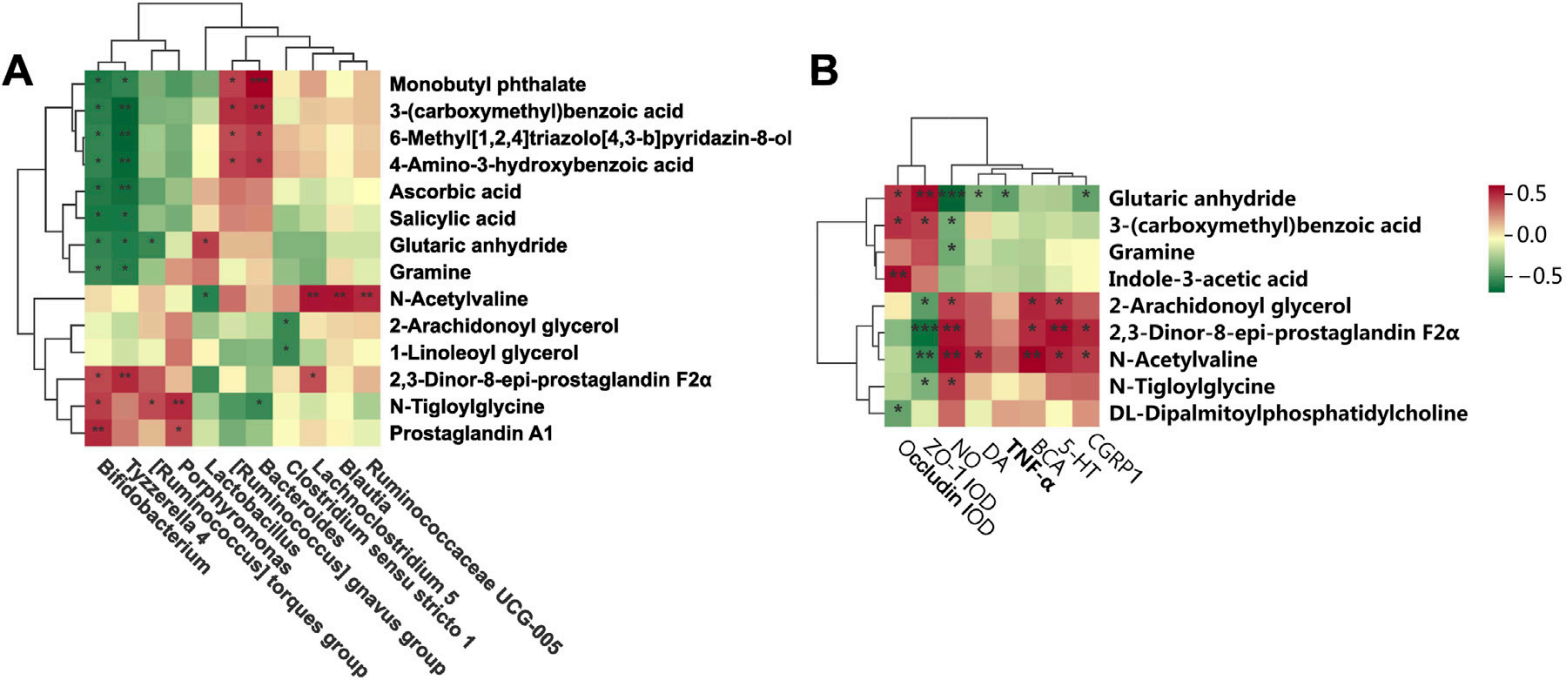
Correlation analysis among gut microbiota, serum metabolites and migraine-related factors. **(A)** Heatmap showing the correlation between gut microbiota and serum metabolites at the genus level. **(B)** Correlation between serum metabolites and migraine-related factors (^*^ *P* < 0.05, ^**^ *P* < 0.01, and ^***^ *P* < 0.001).

Spearman correlation analysis employed to generate a correlation heatmap, elucidating the relationships among serum metabolites and genera, as well as the correlation between microbiota metabolites, as well as between microbiota metabolites and brain tissue migraine-related indices (5-HT, CGPR1, TNF-*α*, IL-1β, and NO, among others), and indicators closely associated with intestinal permeability (ZO-1, Occludin). Figure 8A illustrates distinct correlations among various bacterial genera and metabolites. N-Acetylvaline exhibited a positive correlation with *Lactobacillus*, while 2,3-Dinor-8-epi-prostaglandin F2*α* demonstrated a negative correlation with *[Ruminococcus] gnavus group*. Additionally, Ascorbic acid showed a positive association with *Tyzzerella 4*, and Monobutyl phthalate displayed a negative relationship with *Bacteroides*. Concerning the gut microbiota-migraine connection depicted in Figure 8B, significant effects on the expression of migraine-related factors were observed. Glutaric anhydride positively correlated with NO and TNF-*α*, while 6-Methyl [1,2,4]triazolo [4,3-b]pyridazin-8-ol negatively correlated with ZO-1 AOD. Furthermore, 4-Amino-3-hydroxybenzoic acid exhibited a positive relationship with DA, and Gramine negatively correlated with 5-HT. In conclusion, these findings reveal the complex interplay between fecal metabolite alterations, gut microbiota shifts, and migraine-related factors, highlighting the intricacy of the underlying mechanisms.

## 4 Discussion

Migraine, a complex neurological disorder characterized by recurrent cephalalgia and sensory hypersensitivity, is increasingly associated with dysregulation of the gut-brain axis. Emerging evidence implicates gut microbiota dysbiosis in migraine pathogenesis through altered production of neuroactive metabolites, including short-chain fatty acids (SCFAs) and tryptophan derivatives, which modulate central nociceptive processing and neuroinflammatory cascades (Guo et al., 2019). This study elucidates novel perspectives on the therapeutic mechanisms of Baizhi, a traditional Chinese medicine that has been historically employed for headache management since the Qin-Han Dynasties (Jieyu et al., 2024). We demonstrate that BZ alleviates NTG-induced migraine in rats through a multi-targeted network encompassing neurotransmitter homeostasis restoration, anti-inflammatory modulation, gut microbiota restructuring, and systemic metabolic reprogramming.

BZ significantly attenuated migraine-associated monoamine neurotransmitter imbalances by reducing 5-HT and DA overproduction in plasma and brain tissuesy (Sedat, 2020). This aligns with current anti-migraine strategies targeting CGRP signaling via 5-HT1F receptor activation or direct CGRP receptor antagonism (Vries et al., 2020). Crucially, BZ reduced the abnormal increase of 5-HT in plasma and brain tissue, potentially mitigating neurogenic inflammation and vasodilation underlying migraine aura (Vernieri, Moro, Altamura, et al., 2010). BZ suppression of TNF-*α* and PGE2 suggests inhibition of NF-κB and COX-2 pathways, consistent with its known bioactive metabolites like imperatorin and isoimperatorin.

A growing body of literature confirm that gut microbiota play a causal role to the development and exacerbation of migraine (He et al., 2023). Further analysis from the perspective of gut microbiota function revealed that BZ intervention significantly enriches the abundance of *Lactobacillus* and *Ruminococcus_gnavus_group* in the gut. Enrichment of *Lactobacillus* and *Ruminococcus_gnavus_group* may enhance butyrate production, strengthening epithelial tight junctions and reducing bacterial endotoxin translocation (Hiroshi, 2016).

Both groups of bacteria are important butyrate-producing bacteria in the gut: *Lactobacillus* can produce butyrate through carbohydrate fermentation, while *Ruminococcus_gnavus_group* is capable of synthesizing butyrate using indigestible polysaccharides in the gut. As one of the key short-chain fatty acids (SCFAs) produced by gut microbiota, butyrate not only serves as the main energy source for intestinal epithelial cells to promote their proliferation and repair, but also enhances the expression and assembly of tight junction proteins (such as Occludin and ZO-1) by activating the AMPK signaling pathway, thereby further strengthening the intestinal barrier (Pereira et al., 2025). Meanwhile, butyrate can inhibit the excessive proliferation of Gram-negative bacteria in the gut, reduce the production and translocation of bacterial endotoxins such as lipopolysaccharide (LPS), and consequently lower the level of endotoxins in the peripheral circulation, alleviating the low-grade inflammatory state in the whole body and the central nervous system—and chronic inflammation is a crucial driving factor for the chronicization of migraine and the increase in pain sensitivity. This microbiota remodeling may suppress hypothalamic-pituitary-adrenal (HPA) axis hyperactivity induced by stressors such as sleep deprivation (Chen et al., 2021), BZ may further attenuate stress-induced dysbiosis via vagal nerve modulation or HPA axis regulation.

Untargeted metabolomics revealed BZ’s systemic modulation of arachidonic acid metabolism, unsaturated fatty acid biosynthesis, biotin pathways, and folate-mediated one-carbon metabolism. The modulation of arachidonic acid metabolism by BZ may suppress CGRP1 release via the 2-AG pathway, as demonstrated by the correlation between 2-AG levels and CGRP1 expression. Notably, *Lactobacillus* abundance positively correlated with 2-AG, suggesting microbiota-mediated modulation of fatty acid amide hydrolase activity. Reduction of 2,3-dinor-8-epi-prostaglandin F2α suggests decreased oxidative stress-derived prostanoids and improved blood-brain barrier integrity. Pathway enrichment analysis highlighted BZ’s regulation of tryptophan, phenylalanine/tyrosine, and arginine-proline metabolism. Tryptophan pathway activation promoted 5-HT synthesis, while phenylalanine/tyrosine modulation stabilized catecholamine (DA/norepinephrine) homeostasis. Arginine-proline metabolism alterations, particularly glutaric anhydride accumulation (negatively correlated with nitric oxide [NO]), may attenuate oxidative stress and NO overproduction (Pereira et al., 2023). These findings suggest a “mitochondrial-metabolite-gut axis” mediated by BZ, where *Lactobacillus*-derived metabolites (e.g., indole-3-acetic acid) may enhances redox balance and mitochondrial efficiency, countering neuronal hyperexcitability (RIKEN Center for Integrative Medical Sciences, The Institute of Medical Science, University of Furusawa et al. (2023). The dual mechanism of “metabolic reprogramming-mitochondrial adaptation” involves: Biotin-mediated pyruvate carboxylase activation facilitates tricarboxylic acid (TCA) cycle-oxidative phosphorylation (OXPHOS) coupling to resolve cerebral energy deficits. Folate-dependent one-carbon metabolism may influence migraine chronification through DNA methylation, warranting chromatin immunoprecipitation sequencing (ChIP-seq) validation.

A negative correlation between glutaric anhydride (mitochondrial metabolite) and NO—positively associated with *Lactobacillus* abundance—suggests microbiota-dependent regulation of NO synthesis via oxidative stress pathways. NO, a critical vasodilator and neurotransmitter, participates in cortical spreading depression during migraine aura. Collectively, BZ orchestrates a multi-dimensional therapeutic network targeting neurotransmitter imbalance, neuroinflammation, gut microbiota composition, and metabolic dysregulation. BZ-driven microbial shifts generate bioactive metabolites (e.g., gramine, salicylic acid, prostaglandin A1) that modulate central nervous function through diverse pathways.

While pioneering in linking gut microbiota-metabolome interactions to BZ’s efficacy, several limitations warrant attention. First, sample size constraints (n = 12/group) and inherent limitations of rodent models in recapitulating human chronic migraine phenotypes (Wang et al., 2024). A single dose was selected based on our preliminary finding of a “more significant efficacy with higher doses” trend. However, this design fails to capture dose-response relationships—a key gap for future work. With a 6.3-fold dose conversion ratio between rats and humans (per experimental animal-human equivalent dose tables), follow-up studies should test doses like 3.6 and 10.8 g crude drug/kg to define the effective range, explore dose-dependent mechanisms, and validate our findings. Second, potential 16S rRNA sequencing biases from primer specificity and ribosomal copy number variation. Focus on taxonomic composition without functional microbiome analysis. Future investigations should validate epigenetic regulation via histone deacetylase (HDAC)/p300-CBP inhibitor studies. Explore dual-target therapies combining BZ with CGRP1 monoclonal antibodies. Addressing these gaps may advance personalized migraine therapeutics, bridging molecular insights with clinical translation.

## 5 Conclusion

**FIGURE 9:**
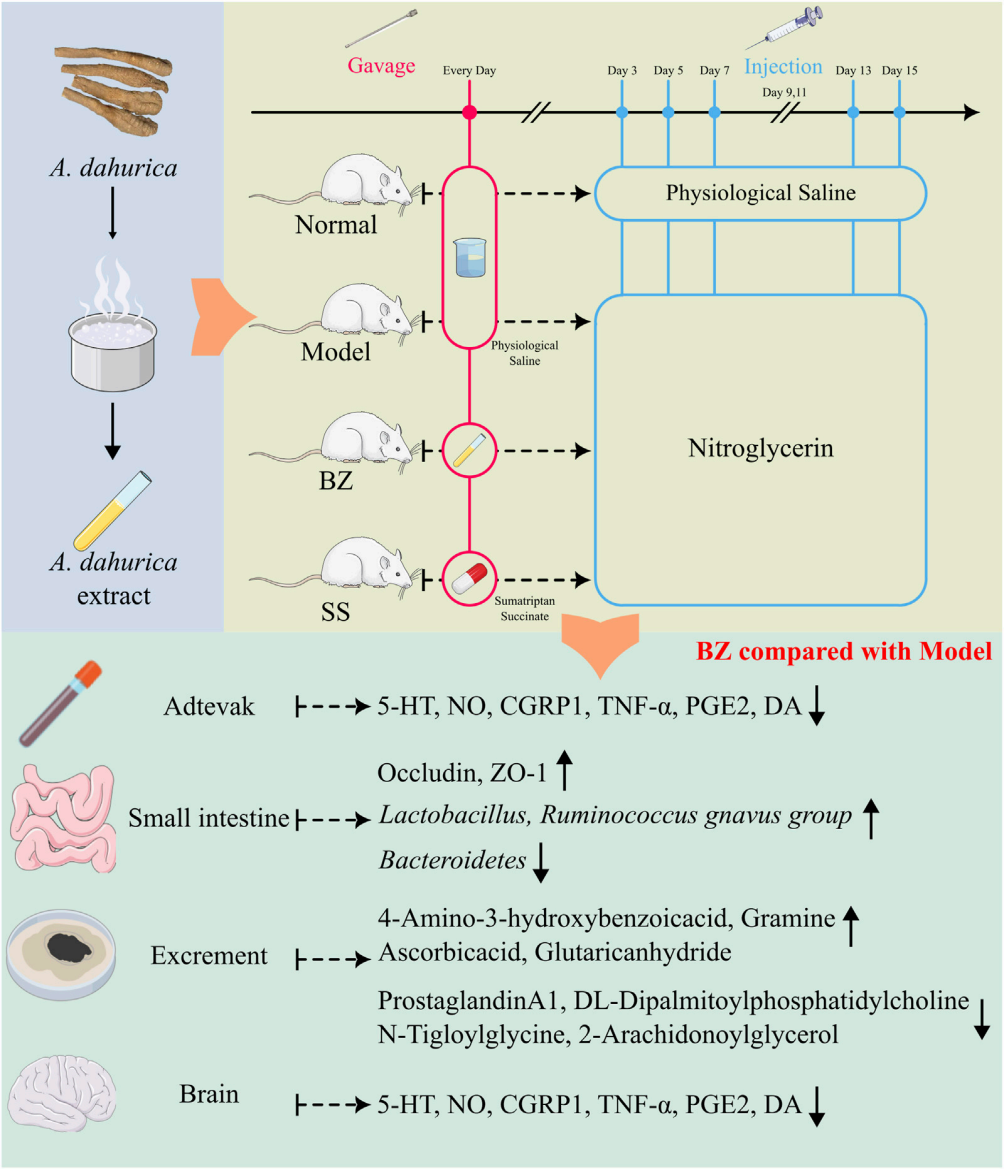
BaiZhi (A. dahurica) extract in migraine relief via gut-brain axis modulation. Using a nitroglycerin-induced rat model (Model group), BZ-treated subjects (oral gavage) demonstrated restored neurotransmitter equilibrium (5-HT/DA balance), suppressed neuroinflammation (reduced TNF-α/PGE2), and enhanced intestinal barrier integrity (upregulated Occludin/ZO-1). Gut microbiota profiling revealed BZ-driven *Lactobacillus* enrichment and metabolic pathway regulation (arachidonic acid/NO axis).

In this study (Figure 9), Baizhi alleviates migraine via gut-brain axis modulation, restoring neurotransmitter balance (5-HT/DA), suppressing neuroinflammation (TNF-α/PGE2), and enhancing intestinal barrier integrity. It remodels the gut microbiota—increasing the abundance of *Lactobacillus* while decreasing that of the *Ruminococcus_gnavus_group*—and regulates mitochondrial-related pathways, such as the arachidonic acid/NO axis. These findings uncover a microbiota-metabolite-brain regulatory network that connects traditional herbology with neuromodulatory mechanisms.

## Data Availability

The data presented in the study are deposited in the Figshare repository. The gut microbiota raw data can be accessed via https://doi.org/10.6084/m9.figshare.29364236, and the serum plasma metabolomics raw data via https://doi.org/10.6084/m9.figshare.29364320. Source data are provided with this paper. Further inquiries can be directed to the corresponding author.
