# Supplementary material for: Multi-omics analysis reveals Angelica dahurica radix extract alleviates migraine in rats via gut microbiota-metabolome-gut-brain axis regulation
Source: Front Pharmacol. 2025 Oct 8;16:1650296. doi: 10.3389/fphar.2025.1650296 (PMC12540402; doi:10.3389/fphar.2025.1650296)
Supplement: Supplementary file 1 [file Supplementaryfile2.doc]

**1. Materials**

**1.1 Instrumentation**

High-performance liquid chromatograph (HPLC) Agilent 1260 Infinity II (USA, Agilent); COSMOSIL C18-MS-II column (100 × 2.1 mm, 1.8 μm); 1:10,000 electronic analytical balance (SQP, Germany, Sartorius Scientific Instruments Co., Ltd.); Milli-Q ultrapure water system Reference (Chengdu Baosaisi Technology Co., Ltd.); 1:100,000 electronic analytical balance (BT125D, Germany, Sartorius Scientific Instruments Co., Ltd.).

**1.2 Test substance**

Byakangelicol (batch number: MUST-17053003, Chengdu Manster Biotechnology Co., Ltd., purity: 99.37%), oxypeucedanin hydrate (batch number: DSTDS012301, Desite Biotech, purity: 98%), and bergapten (batch number: MUST-22080202, Chengdu Manster Biotechnology Co., Ltd., purity: 98.19%). Baizhi was sourced from Baoding, Hebei Province, authenticated as the dried root of *Angelica dahurica*(Fisch.ex Hoffm.)Benth.et Hook.f. by Professor Guihua Jiang (Chengdu University of Traditional Chinese Medicine).The species Angelica dahurica, the botanical drug investigated herein, is covered in the monograph of national pharmacopoeias(Chinese Pharmacopoeia Commission, 2020). After collection, the Baizhi was washed to remove soil and impurities, then dried in an oven at a set temperature of 50 °C until thoroughly dried. Voucher specimens deposited in the university’s herbarium (voucher No. BZ-2024-01, consistent with Sun et al., 2024).

**2 Method and results**

**2.1 Sample preparation**

Take 200 grams of Angelica dahurica (Baizhi) herbs and soak them in ten times their volume of double-distilled water for 1 hour, followed by a 30-minute decoction. After filtering, add water to the filtrate to eight times its original volume and boil again for 30 minutes. Combine both decoctions and concentrate to a concentration of 0.5 grams of crude drug per milliliter. Pass through a 0.45 μm microporous filter membrane, then transfer the remaining filtrate to an injection bottle for 4℃ storage and later use.

#### 2.2 Preparation of standard solution

A mixed standard solution containing Byakangelicol, oxypeucedanin hydrate, and bergaptenwith mass concentrations of 0.144 mg/mL, 0.0424 mg/mL, and 0.014 mg/mL, respectively, was prepared by dissolving in methanol.

#### 2.3. Methodological considerations

**2.3.1 Selection of mobile phase types**

The composition of the mobile phase in liquid chromatography directly determines target analyte responses, peak elution times, and separation efficiency. To evaluate the effects of four mobile phase combinations (A: water-methanol; B: 0.01% formic acid aqueous solution-acetonitrile; C: 0.1% formic acid aqueous solution-acetonitrile; D: 0.01% formic acid aqueous solution-0.01% formic acid methanol solution), we compared signal-to-noise ratios of three analytes under different mobile phase elution conditions using a 10μL mixed standard solution. Results indicated that the 0.01% formic acid aqueous solution-acetonitrile combination demonstrated optimal performance, leading to its selection as the mobile phase.

**2.3.2 Different injection volumes**

While higher sample injection volumes enhance target compound detection sensitivity and improve detection limits, excessive injection may lead to solvent effects in certain analytes and chromatographic column overload causing abnormal peak profiles. This study investigated the elution behavior of target compounds under 1, 2, 5, and 10 μL injection volumes. The results showed that the sample injection amount of 2μL was more suitable.

**2.3.3 Analysis of different detection wavelengths**

Different wavelengths of 254, 300 and 330nm were used for detection. The results showed that the absorbance of 300nm was consistent with the national drug standard YBZ-PFKL-2021004.


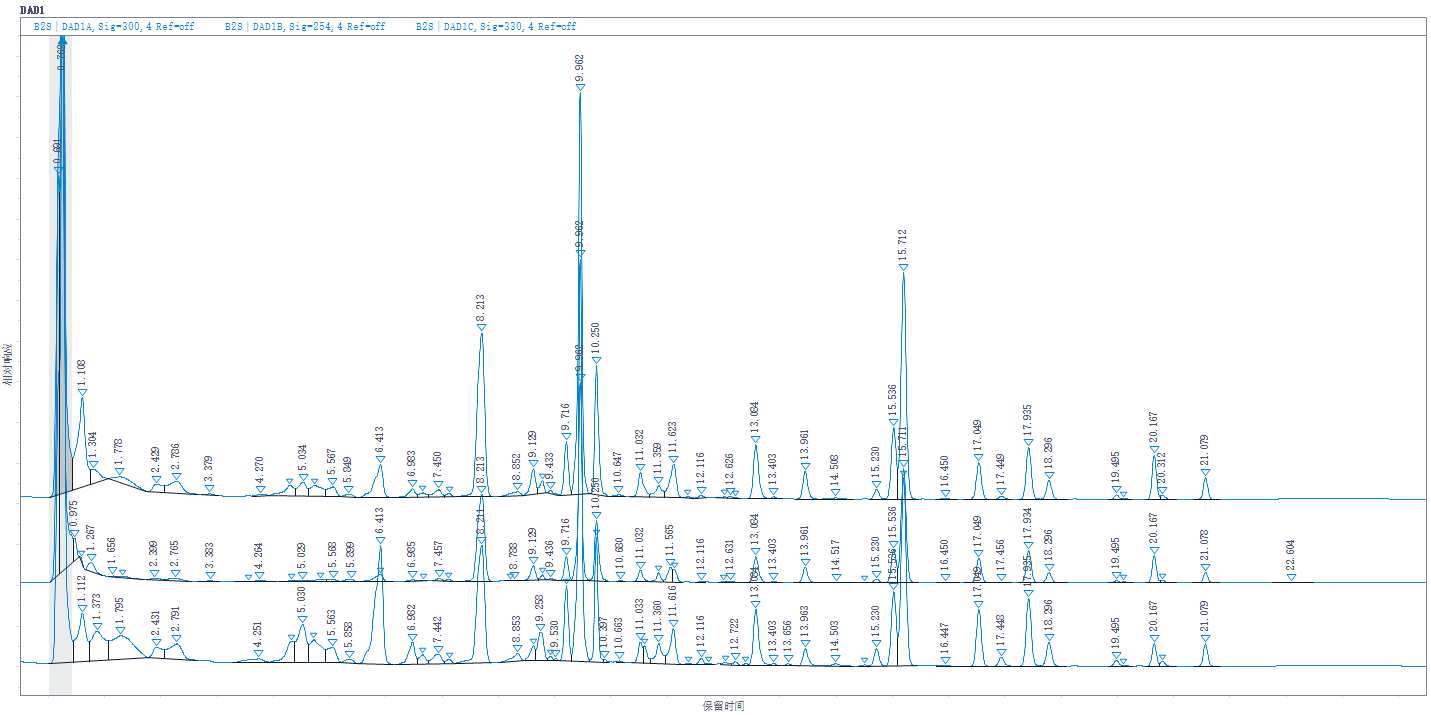


Fig. 1 different detection wavelengths

#### 2.3.4 Chromatographic conditions

A binary mobile phase system consisting of (A) 0.1% formic acid in water and (B) acetonitrile was employed with gradient elution as follows: 0 min (85.0% A, 15.0% B); 5.50 min (72.0% A, 28.0% B); 7.00 min (60.0% A, 40.0% B); 9.50 min (60.0% A, 40.0% B); 16.00 min (50.0% A, 50.0% B); 18.00 min (35.0% A, 65.0% B); 20.00 min (85.0% A, 15.0% B). The flow rate was 0.35 mL/min. The detection was performed at 300 nm.

**2.3.5 Linear relationship examination**

0.1 μL, 0.2 μL, 0.5 μL, 1 μL, 2 μL, 5 μL and 8 μL of the mixed reference substance were precisely absorbed and analyzed by injection respectively. The linear regression was carried out with concentration (X) and peak area (Y), the standard curve was plotted respectively, and the regression equation was calculated.

Table 1 Results of linear relationship investigation

| chemical compound | regression equation | linearity range /μg | ***R^2^*** |
| --- | --- | --- | --- |
| Oxypeucedanin hydrate | y = 8768.6x - 0.9044 | 0.00424-1.424 | 1 |
| Byakangelicol | y = 3412.2x + 0.5851 | 0.0144-1.44 | 1 |
| Bergapten | y = 11883x - 1.8729 | 0.014-1.4 | 1 |


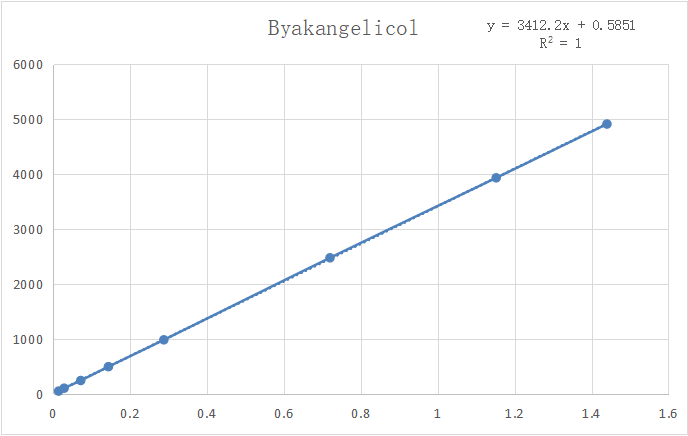

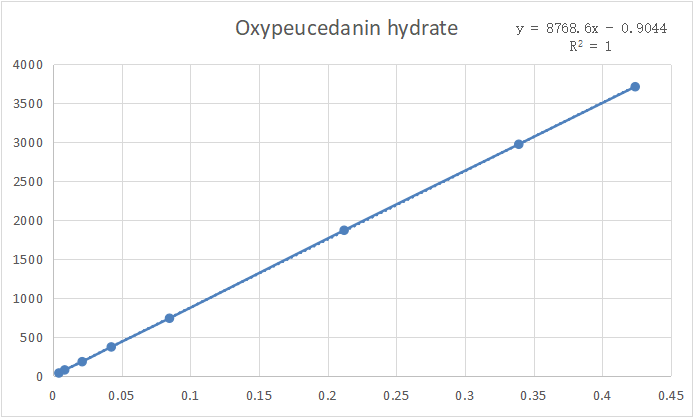

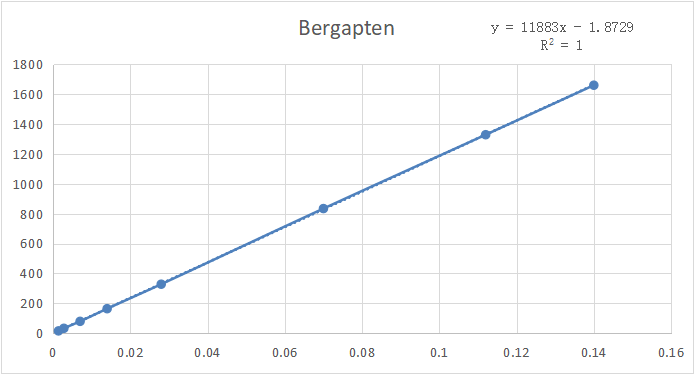


**2.3.6 Precision test** Before selecting oxypeucedanin hydrate chromatographic peak as the reference, the relative retention time RSD of each common peak was 0.03% ~ 0.80%, and the relative peak area RSD was 0.39% ~ 2.39%, indicating that the instrument had good precision.

**2.3.7 Stability test** With oxypeucedanin hydrate chromatographic peak as reference, the relative retention time RSD of each common peak was 0.03% ~ 0.88%, and the relative peak area RSD was 0.37% ~ 2.89%, indicating that the test solution was stable in 7 days.

**2.3.8 Repetition test** With oxypeucedanin hydrate chromatographic peak as reference, the relative retention time RSD of each common peak was 0.01% ~ 0.24%, and the relative peak area RSD was 0.19% ~ 2.49%, indicating that the method had good repeatability.

**2.3.9 Add sample recovery rate test**

Six portions of Baizhi extract samples with known contents (0.5 g each) were accurately weighed. An equal amount of Oxypeucedanin hydrate, Byakangelicol, and Bergapten reference standards were added to each portion to prepare the test solutions, which were then subjected to injection analysis respectively. The results showed that the average spike recoveries of the three chemical components were 93.87%, 95.88%, and 96.93%, respectively. The relative standard deviation (RSD) values of the spike recoveries for each compound ranged from 1.26% to 2.72%, all less than 3%. These findings indicate that the method has good recovery performance.

#### 4. Results of content determination

The chromatogram of the test samples is shown in Figure 1, and the content determination results of the components are shown in Table 1.


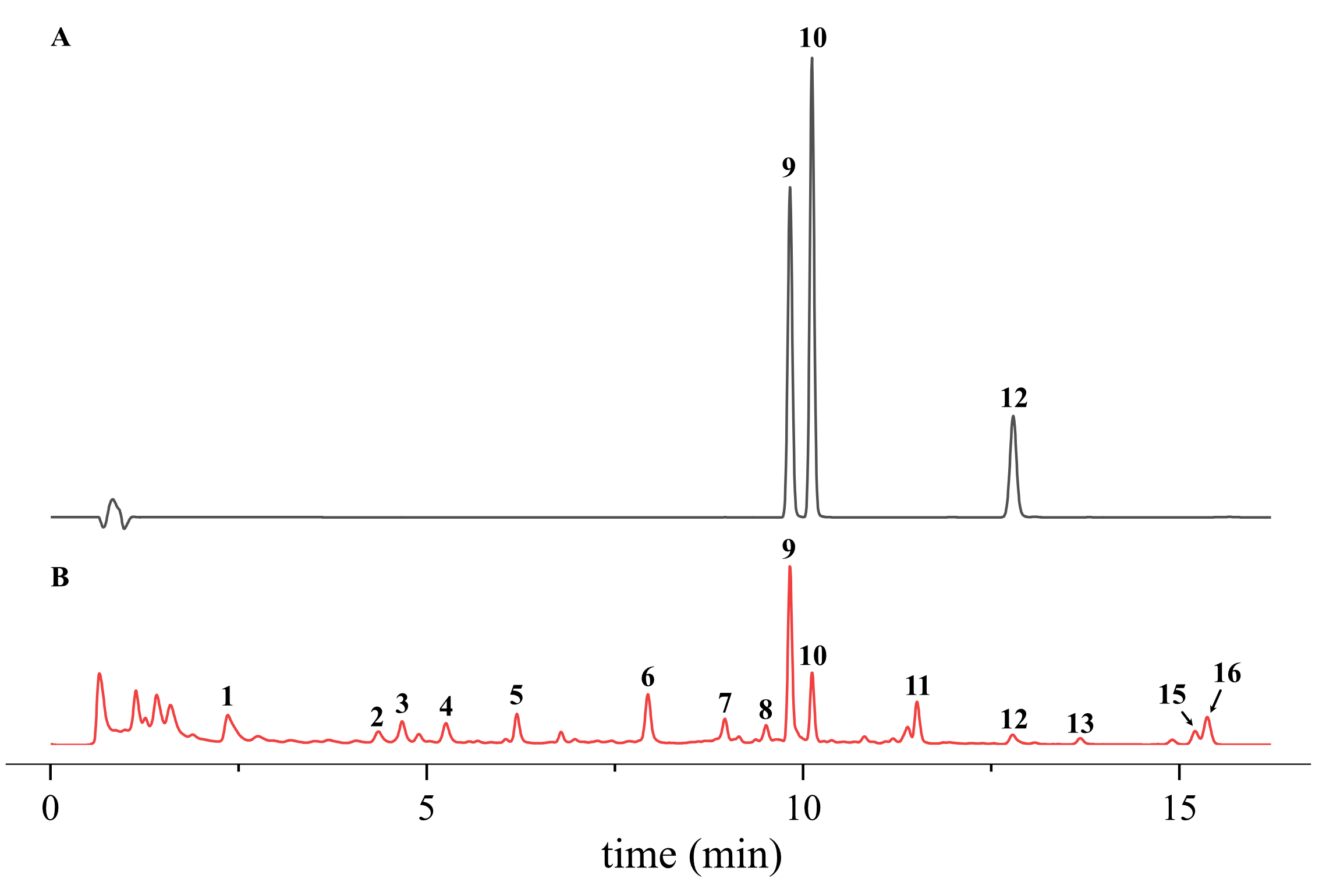


Fig. 1 HPLC chromatograms of mixture standards (A) and test sample (B) of Baizhi

1. Oxypeucedanin hydrate;10.Byakangelicol; 12. Bergapten

**Table 2 Determination results of components and contents of Baizhi samples (mg/g)**

| chemical compound | 1 | 2 | 3 | mean±SD |
| --- | --- | --- | --- | --- |
| Oxypeucedanin hydrate | 0.2183 | 0.2185 | 0.2210 | 0.2193±0.00136 |
| Byakangelicol | 0.2098 | 0.2105 | 0.2102 | 0.2101±0.00036 |
| Bergapten | 0.0152 | 0.0152 | 0.0152 | 0.0152±0.000032 |
