# Supplementary material for: Multi-omics analysis reveals Angelica dahurica radix extract alleviates migraine in rats via gut microbiota-metabolome-gut-brain axis regulation
Source: Front Pharmacol. 2025 Oct 8;16:1650296. doi: 10.3389/fphar.2025.1650296 (PMC12540402; doi:10.3389/fphar.2025.1650296)
Supplement: Supplementary file 2 [file Supplementaryfile1.doc]

**Supplementary Table 1**

**Detailed information on differential metabolites**

| Name | Calc. MW | RT/min | Measured (M/Z) | Normal_vs_Model_VIP | Normal_vs_Model_P-value | Normal_vs_Model_Fold_Change | Normal_vs_Model_Log2FC | Model_vs_BZ_VIP | Model_vs_BZ_P-value | Model_vs_BZ_Fold_Change | Model_vs_BZ_Log2FC | Normal_vs_BZ_VIP | Normal_vs_BZ_P-value | Normal_vs_BZ_Fold_Change | Normal_vs_BZ_Log2FC |
| --- | --- | --- | --- | --- | --- | --- | --- | --- | --- | --- | --- | --- | --- | --- | --- |
| Indole-3-lactic acid | 205.07401 | 9.182 | 206.08152 | 0.750319856 | 0.362383666 | 0.812495252 | -0.299568713 | 1.680925904 | 0.006355862 | 0.581174246 | -0.782957322 | 1.724076253 | 0.003577411 | 0.472201315 | -1.082526034 |
| 4-Amino-3-hydroxybenzoic acid | 153.04272 | 2.209 | 154.05 | 0.377873344 | 0.632875205 | 1.178557738 | 0.237022438 | 1.678300369 | 0.007377231 | 0.395675265 | -1.337611214 | 1.378142784 | 0.027465862 | 0.466326145 | -1.100588776 |
| Prostaglandin A1 | 336.23008 | 14.77 | 335.22273 | 0.82170306 | 0.29979074 | 0.703446742 | -0.507486895 | 1.223427055 | 0.048841788 | 2.068493872 | 1.048580684 | 0.791564765 | 0.269867502 | 1.455075274 | 0.541093789 |
| NP-020750 | 202.02728 | 8.244 | 203.03455 | 1.423618826 | 0.088516559 | 1.813894649 | 0.859090666 | 1.67845902 | 0.008720156 | 0.06055847 | -4.045527425 | 1.615750754 | 0.011548171 | 0.109846685 | -3.186436758 |
| 6-Methyl[1,2,4]triazolo[4,3-b]pyridazin-8-ol | 150.05432 | 2.209 | 151.0616 | 0.254589977 | 0.726879141 | 1.11416826 | 0.155967123 | 1.58957504 | 0.01345078 | 0.425099489 | -1.234127569 | 1.327737153 | 0.033279886 | 0.473632358 | -1.078160446 |
| 2,3-Dinor-8-epi-prostaglandin F2α | 326.20931 | 12.448 | 325.20204 | 2.186772117 | 0.002087231 | 0.419790118 | -1.252259889 | 1.724491318 | 0.002485699 | 2.252988938 | 1.17184023 | 0.148243034 | 0.797870829 | 0.945782492 | -0.08041966 |
| Glutaric anhydride | 114.03072 | 1.91 | 113.02342 | 1.335548198 | 0.13416175 | 2.89487054 | 1.533498832 | 1.693765568 | 0.003912665 | 0.281767227 | -1.827424279 | 0.326250216 | 0.623899183 | 0.815679643 | -0.293925447 |
| 2-Arachidonoyl glycerol | 378.27708 | 25.704 | 379.28436 | 1.78837966 | 0.017749412 | 0.393620926 | -1.345121175 | 1.440784037 | 0.0167382 | 2.382442232 | 1.252441233 | 0.120850835 | 0.847842035 | 0.937779117 | -0.092679942 |
| N-Acetylvaline | 159.08884 | 8.206 | 158.08157 | 2.168945895 | 0.003258093 | 0.472571769 | -1.081394649 | 0.960165857 | 0.113948569 | 1.322796114 | 0.403590713 | 1.638871996 | 0.004320204 | 0.625116099 | -0.677803937 |
| Monobutyl phthalate | 222.08906 | 11.916 | 221.08175 | 0.144269521 | 0.875200993 | 1.021503491 | 0.030694135 | 1.920953052 | 0.000289304 | 0.64099827 | -0.641607632 | 1.649183532 | 0.003755274 | 0.654781971 | -0.610913497 |
| 1-Linoleoyl glycerol | 354.27669 | 26.066 | 355.28397 | 1.341918739 | 0.092282505 | 0.555114126 | -0.84914369 | 1.271327124 | 0.034811251 | 2.241009245 | 1.1641486 | 0.594537701 | 0.37621633 | 1.244015888 | 0.315004911 |
| Ascorbic acid | 176.03139 | 1.814 | 175.02411 | 0.565163269 | 0.490307519 | 1.43404298 | 0.520088264 | 1.502871978 | 0.013274832 | 0.257987991 | -1.954624183 | 1.236888614 | 0.035200532 | 0.369965867 | -1.434535919 |
| Gramine | 129.05807 | 10.593 | 130.06535 | 0.821707632 | 0.329120841 | 1.721824884 | 0.783938423 | 1.223734891 | 0.041014949 | 0.488996351 | -1.032104395 | 0.374794388 | 0.663858149 | 0.841966085 | -0.248165972 |
| Salicylic acid | 138.03098 | 10.046 | 137.0237 | 0.812768115 | 0.296893886 | 1.068171569 | 0.095143391 | 1.633588034 | 0.005505902 | 0.77778839 | -0.362550395 | 1.200676817 | 0.036281975 | 0.830811445 | -0.267407004 |
| N-Tigloylglycine | 157.07309 | 5.278 | 156.06582 | 1.51772743 | 0.059789888 | 0.638827477 | -0.646501729 | 1.769616584 | 0.001602062 | 2.429837349 | 1.280859744 | 1.075053478 | 0.12301428 | 1.552246862 | 0.634358015 |
| DL-Dipalmitoylphosphatidylcholine | 733.56181 | 33.708 | 734.56854 | 0.804952417 | 0.312187079 | 0.785024352 | -0.349190686 | 1.581826994 | 0.002906736 | 3.004461482 | 1.587106426 | 1.474871844 | 0.023040744 | 2.358575429 | 1.23791574 |
